# Gut microbiota-dependent anti-inflammatory mechanisms of berberine in ameliorating hypertension: role of SCFAs, LPS reduction, and STAT3 signaling

**DOI:** 10.3389/fphar.2025.1696934

**Published:** 2026-01-06

**Authors:** Shanshan Wang, Yaqian Hu, Xin Li, Yanhua Ou, Jinling Chen, Yuhan Chen, Jingyi Chen, Kunran Bai, Fangwen Xu, Xingyi Wang, Haoming Du, Difen Yuan, Zhongshan Yang, Jiali Yuan, Haitao Niu

**Affiliations:** 1 Yunnan Provincial Key Laboratory of Integrated Traditional Chinese and Western Medicine for Chronic Disease in Prevention and Treatment, School of Basic Medical Sciences, Yunnan University of Chinese Medicine, Kunming, Yunnan, China; 2 Key Laboratory of Viral Pathogenesis and Infection Prevention and Control (Jinan University), Ministry of Education, School of Medicine, Jinan University, Guangzhou, China; 3 Guangzhou Key Laboratory for Germ-free animals and Microbiota Application, School of Medicine, Institute of Laboratory Animal Sciences, Jinan University, Guangzhou, China; 4 School of Medicine, Jinan University, Guangzhou, China

**Keywords:** gut microbiota, berberine, hypertension, short-chain fatty acids, STAT3

## Abstract

**Background:**

Hypertension is a chronic disease closely related to vascular remodeling, inflammatory response and intestinal flora disorders. Traditional Chinese medicines, especially *Rhizoma Coptidis*, are becoming increasingly popular as a possible cardioprotective drug. Berberine, the main active ingredient of *Rhizoma Coptidis*, has various pharmacological activities, but its specific mechanism of regulating blood pressure through intestinal flora is not clear.

**Methods:**

In this study, the potential targets of berberine were predicted using network pharmacology, and its antihypertensive mechanism was validated in spontaneously hypertensive rats (SHR). A comprehensive evaluation integrating non-invasive blood pressure measurement, echocardiography, histological analyses (H&E and Masson staining), immunohistochemistry, qPCR, metagenomic sequencing, and untargeted metabolomics was performed to investigate the effects of berberine on cardiovascular remodeling, intestinal barrier integrity, gut microbial composition, and metabolic profiles.

**Results:**

Network pharmacology screened 160 common targets of berberine and hypertension, among which STAT3 may play a key role. Animal experiments confirmed that berberine significantly reduced SHR blood pressure and improved aortic fibrosis and cardiac function. In addition, berberine repaired intestinal barrier damage, upregulated ZO-1 and Occludin expression, and significantly altered the structure of the intestinal flora, increasing the abundance of Short-chain fatty acids (SCFAs) - producing bacteria (e.g., *Marvinbryantia, Bacteroides*), while decreasing pro-inflammatory bacteria (e.g., *Mycoplasma, Treponema*). Metabolomics analysis showed that berberine increased fecal SCFAs levels and decreased serum Lipopolysaccharide (LPS). Molecular docking and experimental validation showed that berberine attenuated the inflammatory response by inhibiting STAT3 activation and decreasing colonic IL-6 expression.

**Conclusion:**

Berberine exerts antihypertensive effects by regulating the gut flora-SCFAs-LPS-IL6-STAT3 axis, improving intestinal barrier function, and reducing systemic inflammation. This study provides a new mechanistic basis for berberine treatment of hypertension.

## 1 Introduction

Clinical blood pressure chronically elevated above 140/90 mmHg can be diagnosed as hypertension (Mills et al., 2020), which has increasingly become a risk factor for people’s health as a significant element in the development and advancement of cardiovascular and cerebrovascular disorders (Wu et al., 2022). The pathogenesis of hypertension is multifaceted, involving various systems. Conventionally, it is viewed as a condition affecting two key systems: the renin-angiotensin-aldosterone system (RAAS) and the sympathetic nervous system (SNS), which are crucial for regulating salt and water balance and cardiovascular function (de Kloet et al., 2013; Te Riet et al., 2015), Additionally, new research has identified an immunological mechanism that plays a role in the development and progression of hypertension (Drummond et al., 2019). Factors such as personal lifestyle, geographical and climatic conditions, as well as mental and psychological elements, all influence the onset of high blood pressure (Wang et al., 2023). In the context of modern clinical practice, adapting to contemporary lifestyle changes, such as dietary adjustments and increased physical activity, has proven effective in lowering blood pressure and preventing hypertension (O'Donnell et al., 2023), at present, the clinical treatment of hypertension is dominated by modern medicines, which have the advantages of fast blood pressure lowering speed and clear target organ protection effect (Oparil et al., 2018), but are prone to lead to hepatic and renal damage and part of the combination of medicines is restricted, and long-term use There are some potential hazards (Burnier and Egan, 2019), so the search for drugs to slow down the progression of hypertension from traditional Chinese medicine has become a hot spot of current research.

The human intestine harbors the most populous microbial community within the body, with an estimated 500–1,000 distinct types of gut flora, surpassing the quantity of human body cells by a proportion of 10. This complex microbial ecosystem is intimately linked to the host’s health status (Hou et al., 2022). Current research has pinpointed the function of gut flora and its metabolic byproducts in the onset of various diseases, such as type 2 diabetes mellitus, neurological disorders, cardiovascular disease related to hypertension, and respiratory disorders (Lai et al., 2022; Macpherson et al., 2023; Saxton et al., 2019). Studies have shown that the gut microbiota and gut barrier have attracted much attention in the development and progression of hypertension (Kaye et al., 2020). In both human and animal models (Wang et al., 2023), the composition and dysfunction of the intestinal microbiota have a non-negligible impact on the occurrence and development of hypertension. As research delves deeper, an increasing number of studies indicate that Changes in the gut microbiota’s composition and activity, and the subsequent changes in body metabolism, are intricately connected to hypertension in multiple ways. Research has revealed a significant reduction in gut flora abundance in hypertensive individuals compared to controls. Furthermore, fecal transplants from hypertensive patients to germ-free mice have demonstrated that elevated blood pressure could be transferred through the microbiota, demonstrating the direct effect of gut microbiota on host blood pressure (Gao et al., 2024). For instance, gut microbiota can influence the body’s nervous system (Sharon et al., 2016), immune response (Marques et al., 2017), and inflammatory response (Knauf et al., 2019) thereby affecting hypertension. Pathogenic antigens from the intestinal lumen may penetrate the mucosal lamina propria due to a weak intestinal barrier, resulting in immunological and inflammatory reactions that might exacerbate hypertension disorders (Tang et al., 2017). Investigating the intestinal microecology may offer a novel approach to understanding the pathogenesis of hypertension and its treatment.

*Rhizoma Coptidis* is a commonly used Chinese herbal medicine for the treatment of gastrointestinal and cardiovascular diseases (Feng et al., 2019) and in this study, we screened its main active ingredient as berberine using network pharmacology. Berberine is an isoquinoline alkaloid widely distributed in plants such as *Rhizoma Coptidis* and Huangbai. Modern pharmacological studies have shown that berberine has abundant biological activities, including anti-inflammatory, antioxidant, antibacterial, antiviral, cardiovascular protection, hypolipidemic, hypoglycemic, anti-tumor, anti-ulcer, anti-neurodegenerative, anti-rheumatoid arthritis and so on (Chen et al., 2014). In addition, many of the pharmacological activities of berberine are closely related to its regulatory impacts on the intestinal microbiological community (Habtemariam, 2020).

In this study, spontaneously hypertensive rats were selected to study the effect of intestinal flora on hypertension and to evaluate the efficacy of berberine, to observe the changes in the intestinal microecology of SHR, and to predict potential targets and pathways of berberine in the treatment of hypertension using network pharmacological analyses, which were verified experimentally that berberine modulates the abundance of gut microbiota, increases serum SCFA levels, decreases LPS, and mitigates hypertension through gut microbiota -STAT3 pathway, thereby modulating intestinal inflammation and barrier function.

## 2 Materials and methods

### 2.1 Network pharmacology analysis

#### 2.1.1 Collecting and organizing the main components and potential targets of *Rhizoma Coptidis*

We searched the Traditional Chinese Medicine Systems Pharmacology Database and Analysis Platform (TCMSP) (https://www.tcmsp-e.com/load_intro.php?id=43), and the active ingredients with oral bioavailability (OB) ≥ 30% and drug-likeness (DL) ≥ 0.18 were screened. The PubChem database (https://pubchem.ncbi.nlm.nih.gov/) was searched for each active ingredient (https://pubchem.ncbi.nlm.nih.gov). to retrieve SMILES symbols for each compound. The SwissTargetPrediction online tool (http://swisstargetprediction.ch) predicted the potential targets of these active ingredients based on their SMILES symbols.

#### 2.1.2 Visualization of the ensemble of main targets of xanthium and hypertension-related targets

To identify genes associated with hypertension, the Genecard database (https://www.genecards.org/),OMIM database (https://www.omim.org/) and DisGeNET database (https://disgenet.com/) were searched for the “hypertension” term to screen potential targets for hypertension, and a Wayne diagram was drawn to identify overlapping genes using the Microbiotics online platform (https://www.bioinformatics.com.cn/). Network connections between targets were established using Cytoscape 3.7.1.

#### 2.1.3 Visualization of the ensemble of main targets of xanthium and hypertension-related targets

To identify genes associated with hypertension, the Genecard database (https://www.genecards.org/),OMIM database (https://www.omim.org/) and DisGeNET database (https://disgenet.com/) were searched for the “hypertension” term to screen for potential targets of hypertension, and a Wayne diagram was drawn to identify overlapping genes using the Microbiotics online platform (https://www.bioinformatics.com.cn/). Using the STRING database, protein interaction networks were made, files were downloaded, and core targets were screened and visualized using Cytoscape 3.7.1 based on Closeness unDir ≥ 0.003, Betweenness unDir ≥ 178.76, Degree unDir ≥ 25.85.

### 2.2 Materials and chemicals

From Shanghai Winherb Medical Science Co., Ltd. (Shanghai, China), berberine was acquired. Using high performance liquid chromatography (Agilent ZORBAX SB-C18, 5 μm, 4.6 mm × 250 mm, flow rate: 1.0 mL/min, 280 nm) eluted with MeOH/0.2% HAc-H_2_O (45:55), the purity of berberine (tR = 9.08 min, purity>98%) was confirmed prior to the suggested tests.

### 2.3 Animals and models

Six age-matched male Wistar-Kyoto (WKY) rats and twenty-four 12-week-old male spontaneously hypertensive rats (SHR) that were free of specified pathogens were supplied by Beijing Vital River Laboratory Animal Technology Co., Ltd. (Beijing, China). All of the animals were housed in a standard polypropylene cage that included sterile bedding, a 12-h light/dark cycle, regulated light, humidity, and temperature, as well as free access to food and drink. There was plenty of food and water. All animal methods and procedures have been approved by Jinan University’s Experimental Animal Ethics Committee (approval number IACUC-20210824-02). The rats were acclimated before the trial officially started.

Six WKY rats were used as a blank control group (called group WKY or C) and 18 SHRs were split up into three groups at random (n = 6/group): I. Model group (called group SHR or M); II. Berberine administration group (called group SHR+BB or H); III. Fecal bacteria transplantation group (called group SHR+FMT or F). Berberine administration: berberine was dissolved in sterile saline and administered via oral gavage at a daily dosage of 200 mg/kg; fecal colony transplantation: fecal colony suspension was prepared by resuspending a few grams of donor feces in sterile salin (Shtossel et al., 2023), and was transplanted by oral gavage. An equal dose of sterile saline was administered by gavage to WKY rats as a negative control. The rats were initially put to sleep 24 h following the last dose and then blood was drawn from the aorta. Blood samples were obtained and centrifuged to obtain serum. Subsequently, aorta, colon and feces were collected for subsequent experiments.

### 2.4 Blood pressure measurement

The blood pressure of rats was monitored and recorded twice a week using the tail-sleeve method. Briefly, conscious rats are placed in a fixation box, which is placed on a heating plate at 37 °C for 5 min to acclimatize the rat to the environment in order to stabilize the blood pressure. A transducer is placed in the rat’s tail, and blood flow signals are monitored by inflating and deflating the tail artery while simultaneously applying and releasing pressure, resulting in a blood pressure value. At least 5 measurements are taken to average the blood pressure.

### 2.5 Histological examination

Rat colon and aortic tissues were preserved for 24 h in 4% paraformaldehyde. Sections were implanted and 5 μm. Deparaffinization requires 20 min of xylene I/II, 5 min of 100% ethanol I/II, and 5 min of 75% ethanol, followed by washing. Hematoxylin for 3 to 5 min, followed by rinsing, differentiating, and rinsing again; 85%/95% ethanol for 5 min each; and eosin for 5 min. Dehydration sealing: neutral gum sealing, xylene I/II for 5 min each, and 100% ethanol I/II/III for 5 min each (Aghayev et al., 2022).

Masson staining: After 5–10 min of hematoxylin staining, sections are washed under running water. Following a few minutes of running water washing, the sections are distinguished using 1% hydrochloric acid in alcohol. They are then dyed with an acidic magenta solution for 5 to 10 min and rinsed with distilled water. After roughly 5 min of treatment with a 1% phosphomolybdic acid aqueous solution, counterstain immediately with an aniline blue or green solution for 5 min without washing with water. The slices were exposed to 1% glacial acetic acid for 1 min, then repeatedly dehydrated with 95% alcohol, anhydrous ethanol, xylene, and neutral gum before being mounted.

We used HE staining of rat colon tissues, which can reveal the pathophysiology of the rat colon overall, to examine the impact of berberine on the intestines of SHRs. Additionally, we used Masson staining on rat aorta tissues to analyze the impact of berberine on the blood vessels of SHRs. This made it possible to visually evaluate the level of fibrosis in the rat aorta.

### 2.6 Echocardiography

At the conclusion of the trial, transthoracic echocardiography (Ve-vo2100, Hewlett-Packard, Andover, MA, United States) was used to evaluate cardiac morphology and function in each experimental group. Mask inhalation of isoflurane (2.5% isoflurane inhaler combined with 1 L/min of 100% oxygen) was used to maintain anesthesia. The animals were put on a heating pad in a supine position after having their chest and abdomen shaved. ECG electrodes were used to measure heart rate. The aortic arch and aortic abdomen were the levels at which Doppler pictures were obtained. The time difference between the two readings was used to determine the flow wave’s transmission time from the upper thoracic aorta to the lower abdominal aorta. The average of five separate measurements per rat is represented by each measurement.

### 2.7 Immunohistochemical

To prepare colonic sections for analysis, they were first dewaxed using xylene and then hydrated through a series of ethanol concentrations (70%–100%) following a 2-h incubation in an oven at 65 °C to avoid sample dropout. Subsequently, Epitope retrieval induced by heat was performed on the slides with the pressure cooker method, and 3% H_2_O_2_ was applied for 30 min to inhibit endoperoxidase activity. The sections were then incubated overnight at 4 °C with antibodies, both diluted at 1:100. After a 30-min incubation with horseradish peroxidase-conjugated secondary antibodies, the slides destined for IHC staining were processed with DAB and hematoxylin. The IHC staining observations were seen and photographed with a Leica-M205C microscope. The intensity of the DAB staining was quantified with ImageJ software, and the data were expressed as mean optical density (MOD) values.

### 2.8 Real-time reverse transcriptase polymerase chain reaction analysis

To measure rat intestinal permeability, we employed qRT-PCR to find the expression of genes linked to the integrity of the rat intestinal barrier. Using the Prime Script™ RT reagent Kit with gDNA Eraser from TaKaRa, Japan, total RNA was reverse transcribed after being isolated from colon tissues using the trizol technique. The TB Green™Premix Ex Taq™II Kit was used to set up the qRT-PCR amplification procedure. Following the amplification reaction, a melting curve of the PCR product was created (95 °C for 30 s, 60 °C for 30 s, and 95 °C for 5 s). Gapdh served as the internal reference gene, and the 2^−ΔΔCT^ method was used to measure the gene’s relative expression. The relevant primer sequences are tabulated in Table 1.

**TABLE 1:** The sequences of primers for ZO-1, Occludin, IL-6and GAPDH.

Gene name	Forward primers (5′-3′)	Reverse primers (5′-3′)
ZO-1	ATC​CCA​CAA​GGA​GCC​ATT​CC	TAG​GGT​CAC​AGT​GTG​GCA​AG
Occludin	CAC​TAC​AGC​TTC​CTC​TTG​AC	GTC​TTC​CGG​GTA​AAA​AGA​GT
GAPDH	CCG​GTG​CTG​AGT​ATG​TCG​TG	CCT​TTT​GGC​TCC​ACC​CTT​C
IL-6	GAG​AGG​AGA​CTT​CAC​AGA​GGA​TAC​C	TCA​TTT​CCA​CGA​TTT​CCC​AGA​GAA​C

### 2.9 Analysis of non-targeted histology assays

#### 2.9.1 Metabolite extraction from samples

To extract metabolites, a 1.5 mL centrifuge tube was filled with 100 μL of liquid sample and 400 μL of solution (acetonitrile:methanol = 1:1 (v: v)) containing 0.02 mg/mL of internal standard (L-2-chlorophenylalanine). After 30 min of protein precipitation at −20 °C, samples were cen-trifuged for 15 min at 4 °C (13,000 g). Following a re-dissolution of the samples in 100 µL of solving (acetonitrile: water = 1:1), they were cryo-sonicated for 5 min at 5 °C and 40 kHz, and then centrifuged at 13,000 g at 4 °C for 10 min. After being aspirated, the supernatant was moved to an injection vial equipped with an internal cannula for examination using mass spectrometry.

#### 2.9.2 LC-MS/MS detection

Using a Thermo Fisher Ultra High Performance Liquid Chromatography Tandem Fourier Transform Mass Spectrometer UHPLC-Q Exactive HF-X system, the samples were subjected to LC-MS/MS analysis.

The parameters for mass spectrometry were configured as follows: to achieve comprehensive mass range coverage from 70 to 1,050 m/z, the sample signals were collected in both positive and negative ion modes. The sheath gas pressure was adjusted to 50 psi, and the auxiliary gas flow rate was set to 13 psi, with the auxiliary gas heater kept at a constant temperature of 425 °C. In the positive ion mode, the ion spray voltage was fixed at 3,500 V, while for the negative ion mode, it was set to −3,500 V. Additionally, the ion spray current was regulated at 0.5 mA, and the auxiliary gas current was at 1.5 mA. The ion transfer tube temperature was maintained at 325 °C, and a cyclic collision energy strategy was employed, ranging from 20 V to 40 V and then to 60 V. To ensure the precision and clarity of the data, the resolution was defined at 60,000 for primary mass spectra and 7,500 for secondary mass spectra. Data collection was performed in Data Dependent Acquisition mode, which autonomously selects secondary mass spectra for analysis based on the primary mass spectra outcomes, thereby providing more detailed information.

### 2.10 Macrogenome sequencing and analysis

#### 2.10.1 Constructing DNA library and uploading to the computer

DNA was extracted from mouse feces and then processed using a Covaris ultrasonic crusher to generate 350 bp fragments. Subsequent steps included end repair, addition of A-tails, ligation of junctions, purification, and PCR amplification to create DNA libraries. The prepared libraries were quantified with Qubit 2.0 to confirm a concentration of 2 ng/μL, and the size of the insert fragments was verified using Agilent 2100 Bioanalyzer to ensure compliance with the specifications. Libraries that met the criteria were then accurately quantified by qPCR, requiring a minimum concentration of 3 nM. Once the quality checks were passed, the libraries were diluted to the desired concentration and data volume, mixed accordingly, and subjected to sequencing and analysis on the Illumina PE150 sequencing platform.

#### 2.10.2 Information analysis

Some of the raw data produced by Illumina sequencing is of low quality. These raw data must be pre-processed to provide clean data in order to guarantee the correctness of ensuing analysis. Assembly analysis was performed using MEG-AHIT assembly software, and N-free fragments, or Schaftigs, were obtained by cutting the assembled scaffolds off at N junctions.

To create a non-redundant gene catalogue for the Schaftigs produced by single-sample assembly, fragments shorter than 500 bp were filtered out and then submitted to Meta Gene Mark ORF prediction and CD-HIT de-redundancy. Bowtie2 then compared the Clean Data with the gene catalogue to determine the Unigenes. The Wayne diagram, DIAMOND comparison to the NCBI NR database, and LCA algorithm were utilized to annotate the species information, obtain the abundance of taxonomic hierarchy and gene number, and then conduct additional statistical analysis. Core-pan gene analysis and correlation analysis were also conducted.

### 2.11 Western-blotting

Protein samples were separated by SDS-PAGE electrophoresis, transferred to NC membrane, closed with 5% skimmed milk for 1 h at room temperature, added primary antibody (1:1,000 dilution) and incubated overnight at 4 °C, washed the membrane 4 times by TBST and then added HRP-labeled secondary antibody (1:5,000 dilution) and incubated for 1 h at room temperature, washed the membrane 4 times by TBST, and then ECL was developed to detect the target proteins. Quantitative analysis was performed by ImageJ software.

### 2.12 Statistical analysis

This study followed the principle of reproducibility and was analyzed statistically using GraphPad Prism 9.4.0. The data were presented as mean ± standard error of the mean (mean ± SEM), and comparisons between two groups were made using a two-tailed t-test, and comparisons between multiple groups were made using a one-way analysis of variance. *p* < 0.05 was considered to be statistically significant, *p < 0.05, **p < 0.01, ***p < 0.001, ns: no significant differences.

## 3 Results

### 3.1 Identification of core targets and network analysis between *Rhizoma Coptidis* and hypertension

*Rhizoma Coptidis* was characterized using the TCMSP online platform, and 11 active ingredients were screened, with berberine ranking first. The potential sites of action of these active ingredients in TCMSP were predicted using the SwissTargetPrediction tool, and genes with probability scores greater than 0.1 were screened, resulting in a total of 359 potential targets, and hypertension-related genes were collected from GeneCards, OMIM and DisGeNET databases, totaling 2,175 genes.

**FIGURE 1:**
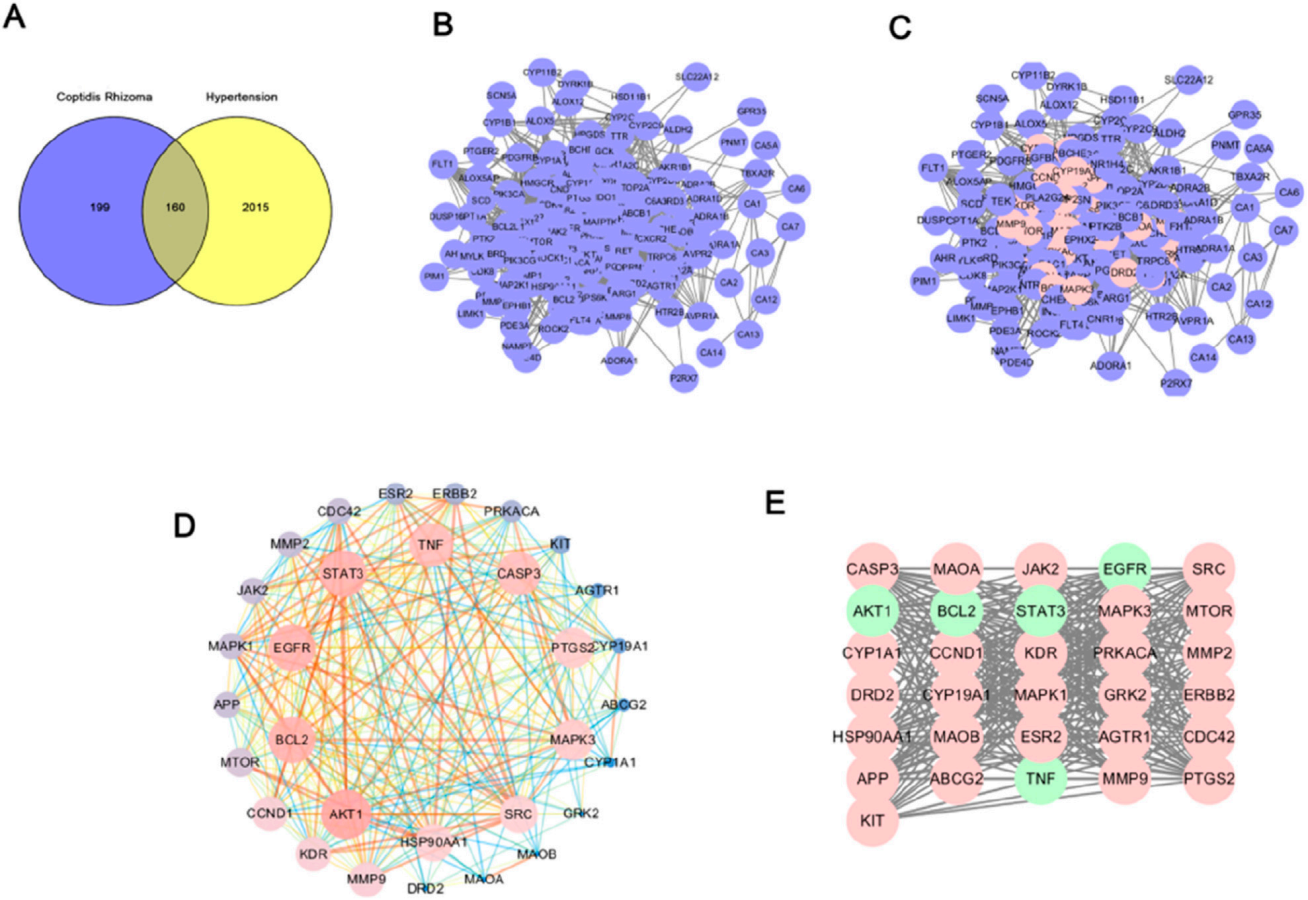
Presence of a hypertensive disease target for *Rhizoma Coptidis* **(A)** Venn diagram of the target of *Rhizoma Coptidis* vs. the target of hypertension; **(B)** Intersecting Targets of *Rhizoma Coptidis* Targets and Hypertensive Targets; **(C)** Core targets in intersecting targets; **(D)** Schematic diagram of core target interconnections; **(E)** Core target visualization.

The results were visualized and analyzed as shown in Figure 1A, and 160 common targets of berberine and hypertension were obtained through the Wayne diagram as shown in Figure 1B, and the core targets were screened out based on Closeness unDir ≥ 0.003, Betweenness unDir ≥ 178.76, and Degree unDir ≥ 25.85 (Figure 1C). Network analysis visualized these parameters (Figure 1D). Combined with protein interaction network analysis, the potential primary targets of berberine in treating hypertension may include AKT1, BCL2, EGFR, STAT3, and TNF (Figure 1E).

### 3.2 Berberine reduces SHR blood pressure and improves SHR cardiovascular structure and function

**FIGURE 2:**
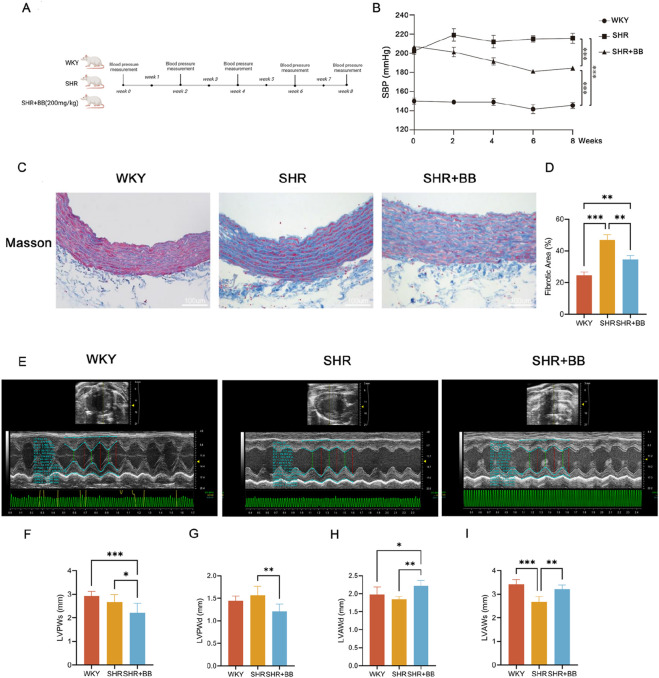
Berberine reduces SHR blood pressure and improves SHR cardiovascular structure and function. **(A)** Intervention and timeline in rats; **(B)** Line graph of blood pressure changes in rats in each group (n = 6, mean ± SD); **(C)** Masson staining of aortic vessels in rats in each group (n = 6, mean ± SD); **(D)** Statistical graph of quantitative analy-sis of masson staining of aortic vessels in rats in each group (n = 6, mean ± SD); **(E)** Small animal ultrasound cardiac imaging in rats in each group (n = 6); **(F)** LVPWs of rats in each group (n = 6, mean ± SD); **(G)** LVPWd of rats in each group (n = 6, mean ± SD); **(H)** LVAWd of rats in each group (n = 6, mean ± SD); **(I)** LVAWs of rats in each group (n = 6, mean ± SD); *P < 0.05, **P < 0.01, ***P < 0.001.

As illustrated in Figures 2A,B, we performed a continuous Berberine intervention on SHR for 8 weeks. During the intervention, the noninvasive tail-collar method was used to monitor blood pressure in rats. The blood pressure value obtained from the initial measurement was used as the basal blood pressure, after which the blood pressure measurements were performed at 2-week intervals. The experimental results showed that Berberine significantly reduced blood pressure in SHR (Figure 2B, *P* < 0.001).

Hypertension is often closely related to vascular lesions (Calvier et al., 2019), in the process of vascular fibrosis, the physiological function of endothelial cells is impaired, which in turn weakens their ability to regulate vascular tension and blood flow (Jandl et al., 2023), at the same time, thickening of the vessel wall, the increase in the number of smooth muscle cells and collagen fibers, as well as damage to the elastic membrane can impair the function of the blood vessels (Heinz, 2021), leading to vascular remodeling, adaptive changes of the vasculature and thus triggering high blood pressure (Dai et al., 2018). We took paraffin-embedded sections of aortic blood vessels from rats and performed Masson staining, and the blue obvious area was the fibrosis-positive area, as shown in Figures 2C,E. The blue area was significantly increased in SHR compared with that in WKY rats (*P* < 0.001); and the blue area was significantly reduced after berberine intervention (*P* < 0.01), suggesting that the degree of fibrosis of aortic blood vessels in SHR was significantly greater than that of WKY, and that berberine significantly reduced the fibrosis of aortic vessels in SHR compared with that of WKY. berberine significantly attenuated the degree of aortic fibrosis in SHR and improved the vascular function of SHR, thus alleviating the hypertension phenotype of SHR.

We used small animal ultrasonography to examine cardiac function in rats, and the results are shown in Figures 2D,F–I. Berberine intervention significantly decreased SHR end-diastolic LV posterior wall thickness (*P* < 0.01) and end-systolic LV posterior wall thickness (*P* < 0.05); berberine intervention significantly increased SHR end-diastolic LV anterior wall thickness (*P* < 0.01) and end-systolic LV anterior wall thickness (*P* < 0.01) at end-diastole and end-systole. These results suggest that berberine can improve the cardiovascular structure and function of SHR.

### 3.3 Berberine reduces the intestinal mucosal barrier damage by regulating SHR intestinal tight junction protein expression

**FIGURE 3:**
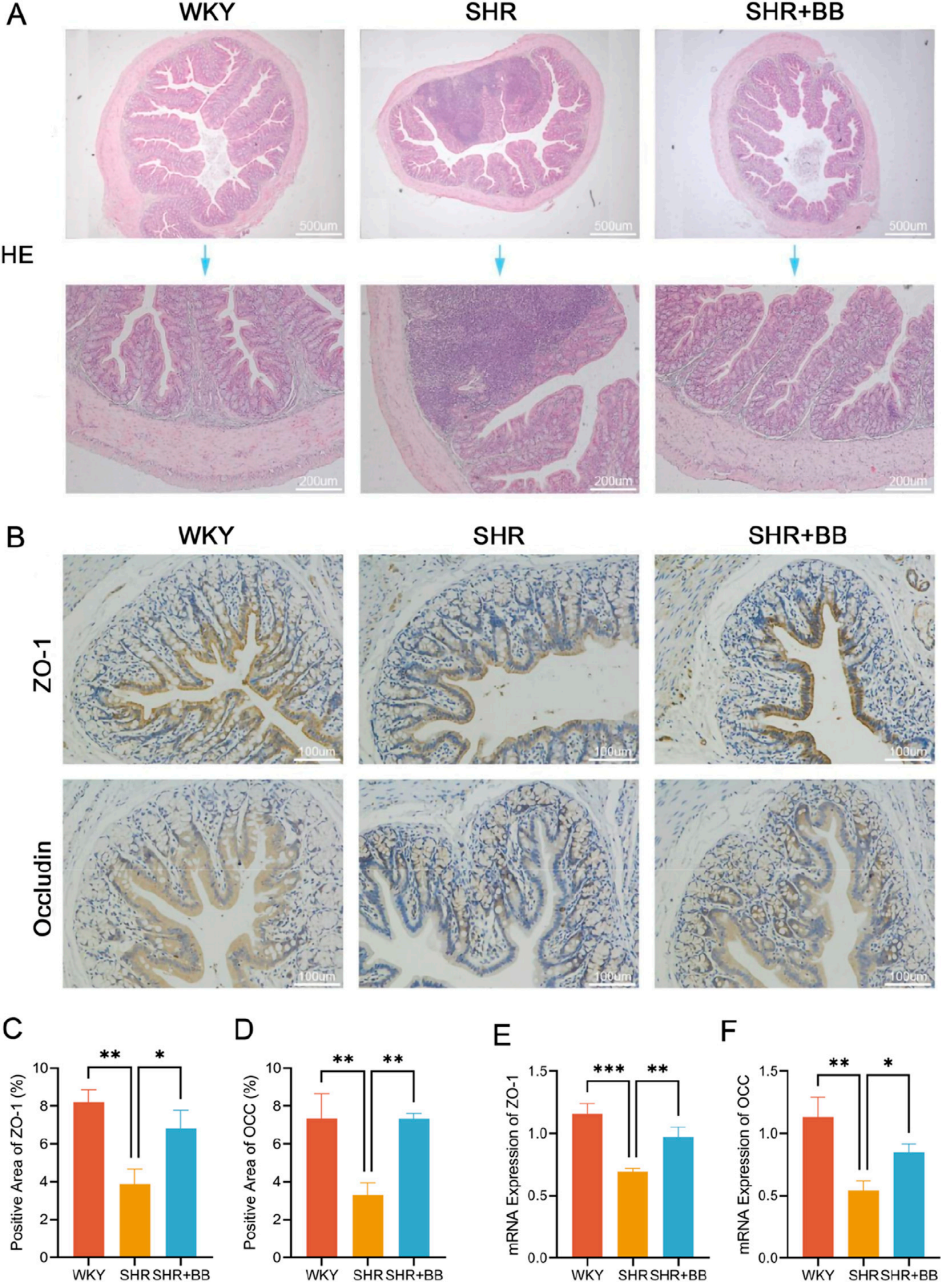
Berberine can repair intestinal barrier damage by regulating the expression of intestinal tight junction proteins. **(A)** HE staining of rat colon in each group (n = 6); **(B)** Immunohistochemical staining of ZO-1 and Occludin in each group (n = 6); **(C)** Quantitative statistic of the positive area for immunohistochemistry of ZO-1 in rat colon in each group (n = 6, mean ± SD); **(D)** Quantitative statistic of the positive area for immunohistochemistry of Occludin in rat colon in each group (n = 6, mean ± SD); **(E)** ZO-1 mRNA expression level in rat colon in each group (n = 6, mean ± SD); **(F)** Occludin mRNA expression level in rat colon in each group (n = 6, mean ± SD). *P < 0.05, **P < 0.01, ***P < 0.001.

Damage to the intestinal mucosal barrier is strongly linked to hypertension (Li et al., 2020). We subjected rat colon to HE staining, and the results showed that the mucosal epithelium of WKY rat colon tissue was intact, the glands were organized nicely, the crypt structure was intact, and there was no infiltration of inflammatory cells; the mucosal epithelium of SHR colon tissue was missing, the crypt structure was damaged, and a significant number of inflammatory cells entered the mucosal layer and the lamina propria; the mucosal epithelial damage was reduced, crypt structure was intact, and inflammatory cell infiltration decreased, and the results indicated that the mucosal damage of SHR colon tissue was reduced by the berberine intervention (Figure 3A). The results indicated that berberine could significantly reduce the intestinal mucosal barrier damage in SHR.

The tight junction proteins occludin and ZO-1 are crucial markers for assessing tissue barrier function and permeability, and intestinal tight junctions are a crucial component of the cytoskeleton that maintains and controls the in-testinal barrier (Wang et al., 2020). ZO-1 (*P* < 0.05) and Occludin (*P* < 0.05) protein expression was lower in SHR rats than in WKY rats, according to immunohistochemical staining of colon tissues and quantitative analysis of protein positive area using ImageJ software. However, following berberine intervention, ZO-1 (*P* < 0.05) and Occludin (*P* < 0.05) were higher in the SHR colon (Figures 3B–D). Levels of mRNA expression of ZO-1 (*P* < 0.001) and Occludin (*P* < 0.05) were significantly lower in the colon of SHR rats than in the colon of WKY rats, according to the qRT-PCR results. Following berberine intervention, the mRNA expression levels of ZO-1 (*P* < 0.05) and Occludin (*P* < 0.05) were restored in the colon of WKY rats (Figures 3E,F). These results suggest that berberine may repair intestinal barrier damage by restoring the expression of SHR tight junction proteins, thereby reducing intestinal permeability.

### 3.4 Berberine modifies the structure of gut microbiota in SHR

**FIGURE 4:**
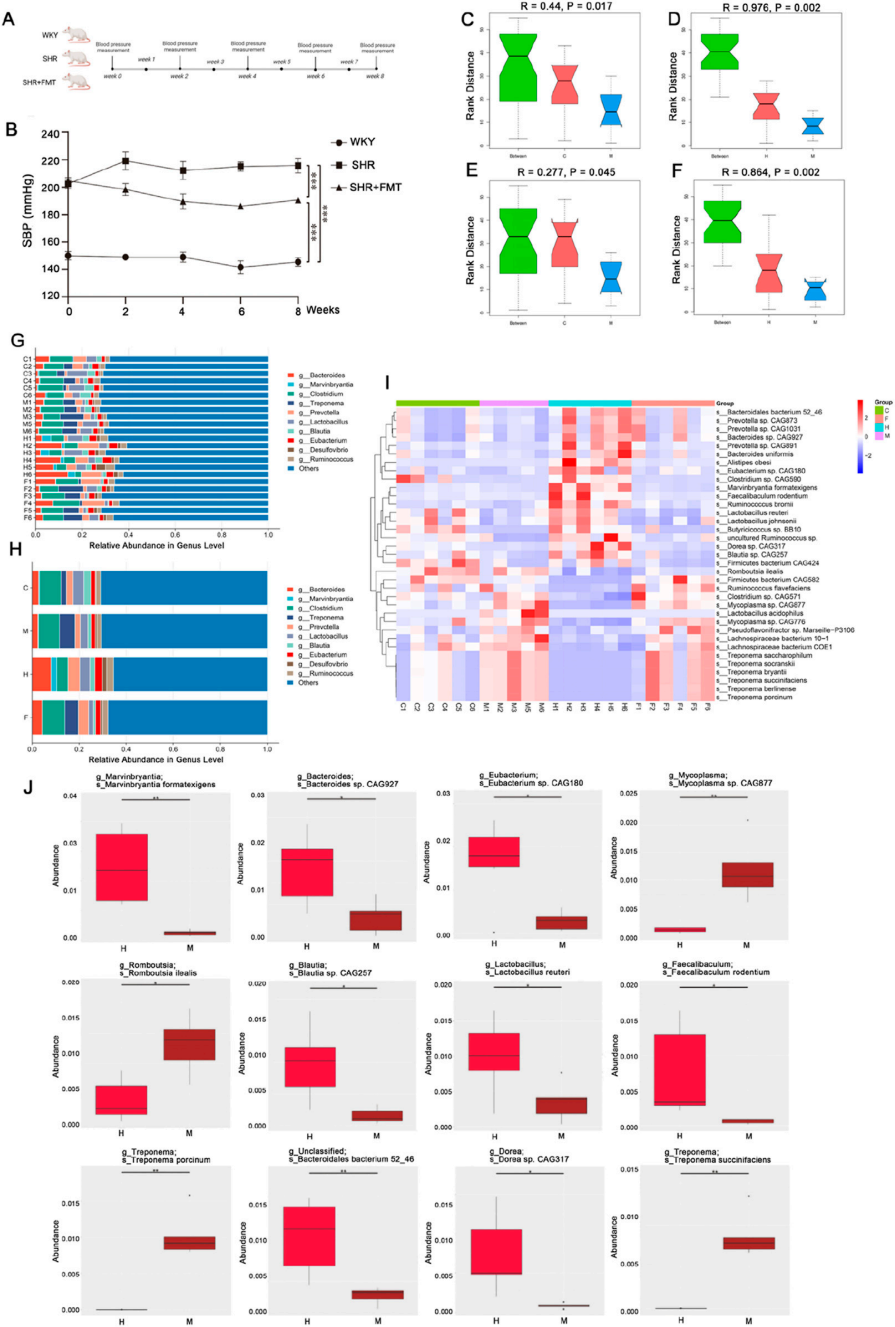
Gut flora is involved in SHR pathogenesis and berberine alters the structure of gut microbiota in SHR. **(A)** Intervention and timeline of rats; **(B)** Line graph of blood pressure changes in rats of each group (n = 6, mean ± SD); **(C)** Anosim analysis graph of group WKY **(C)** vs. group SHR (M) bacterial flora at species level; **(D)** Anosim analysis graph of group SHR+BB (H) vs. group SHR (M) bacterial flora at species level; **(E)** Anosim analysis graph of group WKY **(C)** vs. group SHR (M) bacterial flora at genus level; **(F)** Anosim analysis graph of group SHR+BB (H) vs. group SHR (M) bacterial flora at genus level; **(G)** Statistical bar chart of top10 bacterial genera for a single sample at genus level; **(H)** Histogram of top10 genus statistics of samples of each group at genus level. **(I)** Heat map of significantly different bacterial flora in individual samples of each group; **(J)** Differential bacterial flora in SHR rats (M) after berberine intervention (H) (n = 6, mean ± SD). *P < 0.05, **P < 0.01, ***P < 0.001.

To verify the link between gut microbiota and the development of hypertension, as shown in Figure 4A, we transplanted fecal bacteria from WKY rats into SHR rats. For convenience in the sequencing map, this group is designated as Group F, and the results, as shown in Figure 4B, demonstrated that the SHRs receiving the flora transplant had a markedly lower blood pressure (*P* < 0.001, Figure 4B); Anosim analysis of the gut microbiota of the rats showed significant differences between the groups in the WKY rat (C) and the SHR (M) at the species level and genus level. And the differences also occurred in the SHR (M) and berberine-intervened SHR (H), (Figures 4C–F). Meanwhile, Accordding to the sequencing data, SHR’s gut microbiota had a substantial alteration in structure at the genus level after berberine intervention (Figures 4G,H). Specifically, the relative abundance of *Bacteroides*, *Marvinbryantia*, *Prevotella*, *Blautia*, *Eubacterium*, *Desulfovibrio*, and *Desulfovibrio* was significantly increased after berberine intervention (Figures 4G,H). *Desulfovibrio* and *Ruminococcus*, and significantly reduced by *Clostridium* and *Treponema*; the above results indicated that the pathogenesis of SHR was related to the intestinal flora, and that berberine intervention could significantly change the intestinal microbial structure of SHR.

According to the existing experimental results, we hypothesized that the antihypertensive effect of berberine might be related to the intestinal flora, so we screened the flora with significant changes in SHR after berberine intervention, as shown in the heatmap with significantly different species in Figure 4I, and screened the difference flora from the species level of the difference between each group mainly had 35 species.

MetaStats analysis can compare the species differences contained in the samples of the two groups, in order to more clearly define the relationship between the effect of berberine on SHR and the intestinal flora, We identified the bacterial communities that showed significant differences after BB intervention. As shown in Figure 4J, berberine intervention significantly reduced SHR *g_Mycoplasma;s_Mycoplasma* sp. *CAG877*, *g_ Romboutsia; s_Romboutsia ilealis*, *g_Treponema; s_Treponema porcinum*, *g_Treponema; s_Treponema succinifaciens* strain abundance, and BB intervention significantly elevated SHR *g_ Marvinbryantia; s_Marvinbryantia formatexigens*, *g_Bacteroides; s_Bacteroides* sp. *CAG927*, *g_Eubacterium; s_Eubacterium* sp. *CAG180*, *g_Blautia; s _Blautia* sp. *CAG257*, *g_Lactobacillus; s_Lactobacillus reuteri*, *g_Faecalibaculum; s_Faecalibaculum rodentium*, *g_Unclassified; s_Bacteroidales bacterium 52_46*, *g_Dorea; s_Dorea* sp. *CAG317* strain abundance.

### 3.5 Berberine reduces inflammatory response by increasing the abundance of SCFAs-producing bacteria

**FIGURE 5:**
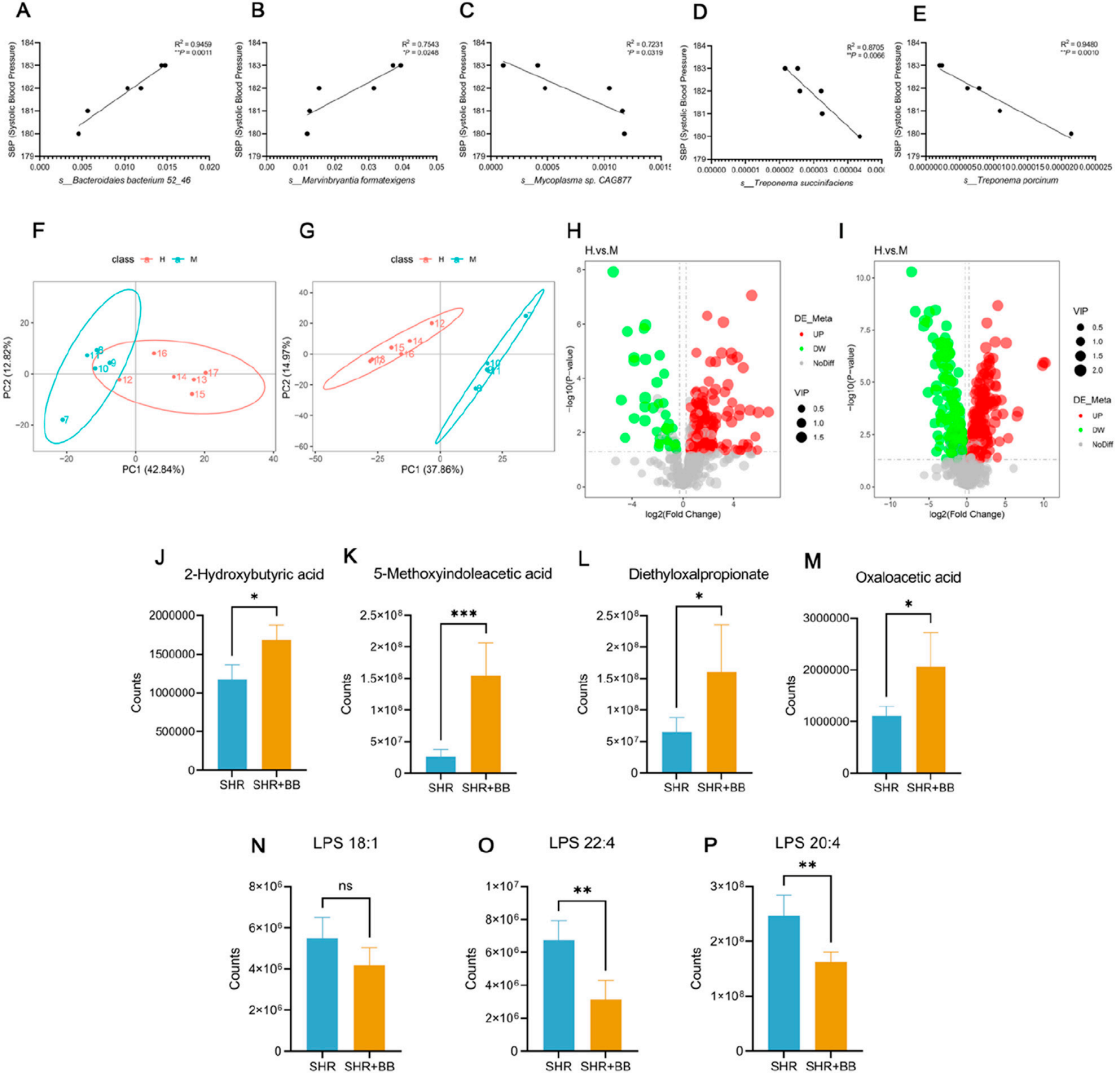
**(A)** Correlation analysis of blood pressure with *s__Bacteroidales bacterium 52_46* (n = 6); **(B)** Correlation analysis of blood pressure with *s__Marvinbryantia formatexigens* (n = 6); **(C)** Correlation analysis of blood pressure with *s__Mycoplasma* sp. *CAG877* (n = 6); **(D)** Correlation analysis of blood pressure with *s__ Treponema succinifaciens* (n = 6); **(E)** Correlation analysis of blood pressure with *s__Treponema porcinum* (n = 6); **(F,G)** Positive and Negative Ion Mode PCA Analysis of Serum Non-Targeted Metabolites in SHR Following Berberine Intervention (n = 6); **(H,I)** Volcano Plot of Differential Metabolites in SHR Serum Following Berberine Intervention in Positive and Negative Ion Modes (n = 6); **(J)** Serum 2-Hydroxybutyric acid levels of SHRs and berberine gavage SHRs (n = 6, mean ± SD); **(K)** Serum 5-Methoxyindoleacetic acid levels of SHRs and berberine gavage (n = 6, mean ± SD); **(L)** Serum Diethyloxalpropionate acid levels of SHRs and berberine gavage (n = 6, mean ± SD); **(M)** Serum Oxaloacetic acid levels of SHRs and berberine gavage (n = 6, mean ± SD); **(N)** Serum LPS 18:1 levels of SHRs and berberine gavage (n = 6, mean ± SD); **(O)** Serum LPS 22:4 levels of SHRs and berberine gavage (n = 6, mean ± SD); **(P)** Serum LPS 20:4 levels of SHRs and berberine gavage (n = 6, mean ± SD). *P < 0.05, **P < 0.01, ***P < 0.001.

We correlated five strains with *P* < 0.01, *s__Marvinbryantia formatexigens*, *s__Mycoplasma* sp. *CAG8*77, *s__Treponema porcinum*, *s__Bacteroidales bacterium 52_*46, *s__ Treponema succinifaciens*, correlation analysis with blood pressure revealed that all five bacterial groups showed strong and statistically significant correlation with blood pressure in rats after berberine intervention (Figures 5A–E).

With the exception of *s__ Mycoplasma mycoides CAG877*, all of them can produce SCFAs, and we performed non-targeted sequencing of rat feces and serum, which showed significant differences in fecal metabolism after berberine intervention (Figures 5F,G), and screened for a variety of differential metabolites (Figures 5H,I). We statistically analyzed the SCFAs screened in the feces and found that berberine increased the content of five short-chain fatty acids and their derivatives, namely, 2-Hydroxybutyric acid, 5-Methoxyindoleacetic acid, Diethyloxalpropionate, Oxaloacetic acid, in the SHR (Figures 5J–M).

In addition, we evaluated the changes of LPS in rat serum, and three structures of LPS were detected by non-targeted sequencing, in which berberine significantly reduced LPS 22:4 and LPS 20:4 (*P* < 0.01, Figures 5O,P), but had no effect on LPS 18:1 structure (Figure 5N). The above results suggest that berberine can reduce inflammatory responses by increasing the abundance of short-chain fatty acid-secreting strains and decreasing endotoxin LPS entry into the bloodstream.

### 3.6 Berberine decreases blood pressure via the gut bacteria-IL6-STAT3 axis

**FIGURE 6:**
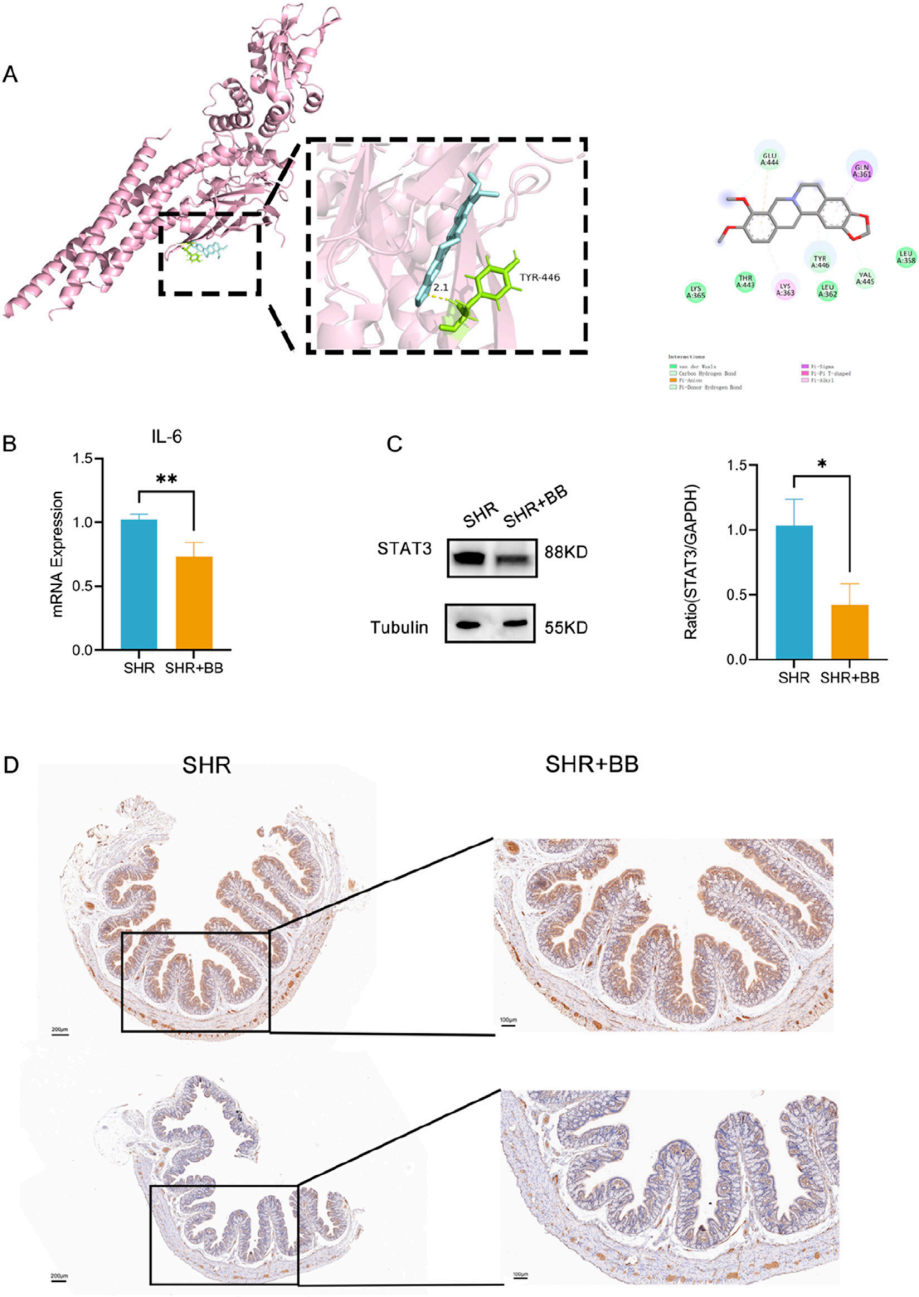
**(A)** Schematic diagram of STAT3 docking with berberine molecule; **(B)** Gut IL-6 mRNA expression levels in rats of each group; **(C)** Schematic representation of STAT3 expression levels in the colon of rats in each group; **(D)** Immunohistochemical results of STAT3 in the rat colon. *P < 0.05, **P < 0.01, ***P < 0.001.

**FIGURE 7:**
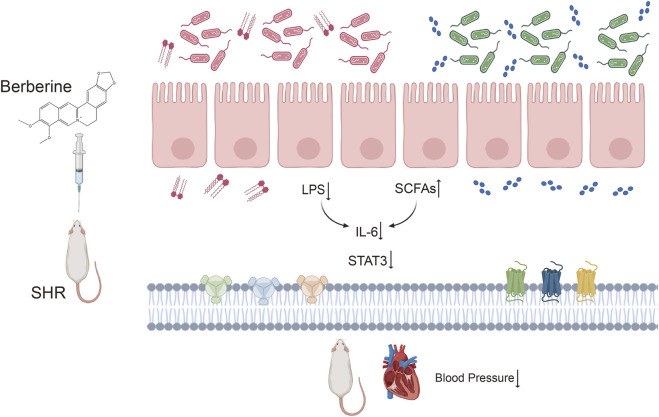
Berberine alleviates inflammation and hypertension by increasing short-chain fatty acids and reducing LPS through its core mechanism of inhibiting the IL-6-STAT3 pathway.

Through metabolomics analysis, we found that berberine significantly reduced the levels of lipopolysaccharide (LPS) in the serum of spontaneously hypertensive rats (SHR) and alleviated intestinal inflammation. LPS, a major component of the outer membrane of Gram-negative bacterial cell walls, is one of the key pathogenic factors. Based on the results of target screening through network pharmacology and *in vivo* experimental data, we propose the hypothesis that berberine may regulate STAT3 by modulating the gut microbiota, thereby lowering blood pressure. Our molecular docking analysis revealed that berberine could form hydrogen bonds with the TYR-446 site of STAT3, with a docking energy of −6.11 kcal/mol (Figure 6A). This result was further validated by Western blot experiments, which showed that berberine inhibited the expression of STAT3 in the intestinal environment (Figure 6C, *P* < 0.05). Additionally, qPCR analysis of IL-6 levels in the colon tissue of rats, as IL-6 is an upstream signaling molecule of STAT3, demonstrated that berberine significantly decreased the mRNA expression of IL-6 in the rat colon (Figure 6B, *P* < 0.01). Immunohistochemical staining further confirmed that berberine reduced the expression of STAT3 in the intestine of SHR (Figure 6D). Berberine increases the abundance of SCFA-producing bacteria while decreasing the abundance of Gram-negative bacteria, thereby reducing LPS endotoxins in the blood. Through the gut microbiota-IL6-STAT3 axis, berberine alleviates inflammation and lowers blood pressure (Figure 7).

## 4 Discussion

Accumulating evidence suggests that BB significantly ameliorates the pathogenesis of hypertension (Młynarska et al., 2024). Given the poor oral bioavailability of BB, this study investigated whether its antihypertensive effects are mediated through modulation of the gut microbiota and its metabolites. We confirmed that BB markedly reduced blood pressure in SHR. Furthermore, transplantation of fecal microbiota from normotensive WKY rats into SHR resulted in a substantial reduction in blood pressure, indicating a pivotal role of gut microbial composition in the pathogenesis of hypertension.

Metagenomic and metabolomic analyses revealed that BB treatment significantly increased the relative abundance of *Bacteroides*, *Marvinbryantia*, *Prevotella*, *Blautia*, *Eubacterium*, and *Ruminococcus*, while markedly decreasing *Mycoplasma* and *Treponema*. The reduction in *Treponema* and *Mycoplasma*—genera associated with intestinal and vascular inflammation—may contribute to the protective effects of BB. In addition, BB administration enhanced SCFA-related metabolites, including 2-hydroxybutyric acid, 5-methoxyindoleacetic acid, diethyl malonic acid, and oxaloacetic acid. Notably, BB-upregulated bacterial taxa were strongly correlated with increased SCFA production. Certain *Prevotella* species, for instance, produce succinate and acetate as primary fermentation products, which have been implicated in delaying the onset of hypertension (Ge et al., 2024; Malanca et al., 2024). Similarly, *Blautia* species generate butyrate, acetate, and succinate, which alleviate inflammation and metabolic disorders and are inversely correlated with visceral fat accumulation—a biomarker for obesity-related cardiovascular risk (Shibata et al., 2023). *Eubacterium* is also a key butyrate producer and contributes to intestinal barrier integrity, glucose and cholesterol homeostasis, and immune regulation (Panwar et al., 2021). Consistently, our results indicate that BB promotes butyrate synthesis and metabolism in SHR. Butyrate, along with acetate and propionate, accounts for approximately 80% of gut-derived SCFAs. Although hypertensive individuals often exhibit elevated fecal SCFA levels, their circulating butyrate concentrations are lower than in normotensive subjects. SCFA supplementation has been reported to reduce blood pressure, and butyrate in particular exerts vasoprotective effects through its anti-inflammatory properties (Mann et al., 2024; Tian et al., 2022). Inflammation induced by factors such as diabetes and dyslipidemia can activate endothelial cells and promote hypertension (Park and Park, 2015). SCFAs are absorbed through the intestinal epithelium and can activate vagal afferent fibers, thereby contributing to blood pressure regulation (Dalile et al., 2019).

An interesting question arising from our findings is whether fecal microbiota from BB-treated SHR would confer similar antihypertensive effects when transplanted into untreated SHR. Although this experiment was not performed in the present study, it represents a promising direction for future investigation. Given the pronounced remodeling of gut microbial composition following BB treatment, it is plausible that fecal transplantation from BB-treated donors could transfer beneficial microbial traits to hypertensive recipients and reduce blood pressure. Such experiments would provide more direct evidence that the antihypertensive effects of BB are at least partly mediated by gut microbiota, rather than solely by its direct pharmacological actions.

The inhibition of STAT3 activation by BB appears to involve both direct and indirect mechanisms. Molecular docking analysis demonstrated that BB can directly interact with the STAT3 protein via hydrogen bonding and hydrophobic interactions, potentially interfering with its phosphorylation and transcriptional activity in intestinal tissues. In parallel, our findings and previous reports indicate that BB exerts profound regulatory effects on gut microbiota composition and associated inflammatory signaling. By enhancing SCFA-producing bacteria and reducing endotoxin-producing Gram-negative taxa, BB mitigates intestinal inflammation and downregulates IL-6–mediated JAK/STAT3 pathway activation. Our data suggest that BB lowers blood pressure in SHR through modulation of the gut bacteria–IL-6–STAT3 axis. Therefore, the inhibitory effects of BB on STAT3 likely result from a dual mechanism—both direct binding to STAT3 and indirect suppression via microbiota-mediated anti-inflammatory effects. This dual regulatory pattern may contribute to the restoration of gut homeostasis and attenuation of hypertension-associated inflammation (Zhou et al., 2025).

Nevertheless, the relationship between BB’s antihypertensive effects and its modulation of gut microbiota is not universally consistent across studies (Rikeish et al., 2025). Several reports have clearly demonstrated that BB exerts its beneficial effects through microbiota-dependent mechanisms. For instance, BB has been shown to ameliorate vascular dysfunction in hypertension by downregulating the TMAO–endoplasmic reticulum stress pathway through gut microbiota modulation (Zhuang et al., 2024). Other studies similarly confirmed that BB reduces trimethylamine-N-oxide (TMAO) levels and improves vascular function in angiotensin II–induced hypertensive mice, further emphasizing the central role of gut microbiota in BB’s antihypertensive mechanism.

Conversely, some studies have focused on microbiota-independent mechanisms, highlighting BB’s direct effects on specific enzymes, receptors, or signaling pathways (An et al., 2025). Although BB exhibits low oral bioavailability, its active metabolites or local intestinal actions may elicit pharmacological effects independent of microbiota alterations. For example, BB may directly target vascular smooth muscle or endothelial cells, modulating vascular tone and reducing blood pressure through direct pharmacological mechanisms (Wang et al., 2024). Therefore, while gut microbiota modulation plays a critical role, BB’s antihypertensive effects likely result from an interplay of both microbial and host-mediated mechanisms.

## 5 Conclusion

In the present study, the mechanism of the antihypertensive effect of berberine and its “intestinal-cardiovascular axis” regulatory pathway were revealed by the combined use of network pharmacology prediction and animal experimental validation. The network pharmacology analysis revealed that berberine may act through multiple targets such as STAT3 and AKT1, suggesting that these targets may jointly mediate the multi-target antihypertensive effect of berberine.

In the spontaneous hypertensive rat (SHR) model, we observed a significant cardioprotective effect of berberine. After 8 weeks of intervention treatment, it significantly reduced blood pressure and improved cardiovascular function in hypertensive rats.

In-depth study revealed that berberine regulates the “gut-cardiovascular axis” through multiple pathways: 1. repairing the intestinal barrier function and up-regulating the expression levels of tight junction proteins ZO-1 and Occludin; 2. remodeling the structure of the intestinal flora, and significantly increasing the abundance of SCFAs-producing bacteria, such as Marvinbryantia and *Bacteroides*; and 3. improving the 2-hydroxybutane in the feces, which is a key component of the cardiovascular function. Levels of multiple SCFAs in feces while decreasing serum levels of the pro-inflammatory factor LPS. Molecular docking and experimental validation showed that berberine significantly inhibited IL-6-STAT3 signaling pathway activation.

## Data Availability

The original contributions presented in the study are publicly available. This data can be found here: Sequence Read Archive (SRA) repository, accession number PRJNA1381256.

## Abbreviations

BB, berberine; SHR, spontaneously hypertensive rats; WKY, Wistar-Kyoto; RAAS, renin-angiotensin-aldosterone system; SNS, sympathetic nervous system; H&E, hematoxylin and eosin; ECG, electrocardiogram; DAB, diaminobenzidine; MOD, mean optical density; IHC, immunohistochemical; qRT-PCR, real-time reverse transcriptase polymerase chain reaction; UA, uric acid; XOD, xanthine oxidase; SBP, systolic blood pressure; LVPWd, left ventricular posterior wall diastole; LVPWs, left ventricular posterior wall systole; LVAWd, left ventricular anterior wall diastole; LVAWs, left ventricular anterior wall systole; KEGG, Kyoto Encyclopedia of Genes and Genomes; PCA, principal component analysis; VIP, variable importance in the projection; SCFA, Short-chain fatty acids; LPS, Lipopolysaccharide.
